# Integrated transcriptome and metabolome analysis unveils the mechanism of color-transition in *Edgeworthia chrysantha* tepals

**DOI:** 10.1186/s12870-023-04585-1

**Published:** 2023-11-16

**Authors:** Ningzhi Zhou, Yujuan Yan, Yafeng Wen, Minhuan Zhang, Yu Huang

**Affiliations:** 1 College of Landscape Architecture, Central South University of Forestry and Technology, Changsha, 410004 China; 2 Hunan Big Data Engineering Technology Research Center of Natural Protected Areas Landscape Resources, Changsha, 410004 China; 3 Nanning University, Nanning, 530200 China

**Keywords:** *Edgeworthia chrysantha*, Flower color change, Carotenoid, Metabolome, Transcriptome

## Abstract

**Background:**

*Edgeworthia chrysantha*, a deciduous shrub endemic to China, is known for its high ornamental value, extensive cultivation history, and wide-ranging applications. However, theoretical research on this plant is severely lacking. While its flowering process displays striking color transitions from green (S1) to yellow (S2) and then to white (S3), the scientific exploration of this phenomenon is limited, and the underlying regulatory mechanisms are yet to be elucidated.

**Results:**

Correlation analysis between phenotypic measurements and pigment content revealed that carotenoids and chlorophyll are the key pigments responsible for the color changes. Metabolomic analysis of carotenoids demonstrated that lutein and β-carotene were present at higher levels in S1, while S2 exhibited increased diversity and quantity of carotenoids compared to other stages. Notably, antheraxanthin, zeaxanthin, lycopene, and α-cryptoxanthin showed significant increases. In S3, apart from the colorless phytoene, other carotenoid metabolites were significantly reduced to extremely low levels. Transcriptomic data indicated that *PSY*, *Z-ISO*, *crtZ*, *ZEP*, *PDS* and *ZDS* are key genes involved in carotenoid biosynthesis and accumulation, while *NCED* plays a crucial role in carotenoid degradation. *SGR* was identified as a key gene contributing to the progressive decline in chlorophyll content. Additionally, three transcription factors potentially regulating carotenoid metabolism were also identified.

**Conclusions:**

This study represents the first systematic investigation, spanning from phenotypic to molecular levels, of the color-changing phenomenon in *E. chrysantha.* The study elucidates the crucial pigments, metabolites, genes, and transcription factors responsible for flower color changes during the flowering process, thereby providing preliminary understanding of the intrinsic regulatory mechanisms. These findings establish a theoretical foundation for the genetic improvement of flower color in *E. chrysantha*.

**Supplementary Information:**

The online version contains supplementary material available at 10.1186/s12870-023-04585-1.

## Background

Flower color is an important ornamental trait and breed classification criterion for plants [1, 2], which not only enhances the ornamental, application, and economic values of flowers [3–6], but also serves as a crucial functional trait for plants to transmit signals to pollinators, playing a significant role in plant reproduction [7, 8]. Flower color in plants is the result of the interplay of various factors, among which the types, contents, and spatiotemporal combinations of pigments play a decisive role [9–11]. In nature, the diversity of plant flower colors largely depends on the differential expression of key enzyme-coding genes in the pigment biosynthetic pathway in different spatiotemporal contexts [12–14].

Carotenoids, the second major class of pigments in plants, are ubiquitously present in nature and typically exist in crystalline or precipitated form in plastids of cells [15]. With multiple conjugated double bonds, carotenoids can absorb light in the 400-500 nm range, conferring yellow, orange, and red hues to many plants [16, 17]. In addition to their involvement in various biological processes in plants, including photosynthesis, photomorphogenesis, photoprotection, and plant development [18], carotenoids also serve as precursors for the biosynthesis of plant growth regulators such as abscisic acid (ABA) and other hormones, which are crucial for plant growth and development [19, 20]. In ornamental flowers, lutein is the key metabolite that determines the color of marigold (*Tagetes erecta*). Due to its abundant content, marigold is often used as a plant material for extracting lutein [21]. Chrysanthemum (*Chrysanthemum morifolium*) has a wide variety of cultivars with diverse flower colors. Research showed there is no significant difference in the expression level of carotenoid synthesis genes between the petals of white and yellow cultivars, but the expression level of degradation enzymes in the petals of white cultivars is significantly higher than that of yellow cultivars. By inhibiting the expression of degradation enzyme genes, the color of petals can be changed from white to yellow [22]. Furthermore, some transcription factors can indirectly affect the biosynthesis of carotenoids in plant tissues by regulating fruit ripening; however, most of the relevant reports are based on research on model plants, and there are few reports on transcription factors regulating carotenoid synthesis specifically in floral organs [23, 24]. Therefore, identifying carotenoid metabolites closely associated with color phenotypes in floral organs, clarifying the biosynthetic pathway and regulatory mechanism of carotenoids in floral organs, and identifying key regulatory structural genes and transcription factors is of great significance for the development of economic value, improvement of flower colors, and breeding of new varieties in ornamental plants.

*Edgeworthia chrysantha* Lindl. is a deciduous shrub belonging to the genus Edgeworthia of the Thymelaeaceae family, endemic to China [25]. The flowers bloom before the leaves, with a flowering period lasting up to 2 months in late winter and early spring. During the flowering process, the tepals of *E. chrysantha* exhibit a noticeable color change phenomenon. They are green during the bud stage, turn bright yellow during the peak blooming period, and fade to white towards the end of the blooming period. These different colored tepals coexist on a single inflorescence, making it highly ornamental and valuable for research. Additionally, due to its remarkable adaptability, easy cultivation, and fragrant aroma, *E. chrysantha* is widely utilized in landscapes and has a long history of cultivation dating back to the Han Dynasty (202 BC–220 AD) [26]. However, academic research pertaining to this species remains limited, with fewer than a hundred relevant papers published, most of which are dated. In particular, studies exploring the color change phenomenon of *E. chrysantha* is almost non-existent, with only Ono et al*.* previously studying it from the perspective of pigments. They measured the flavonoid content at three stages of the color change process and found no significant differences in flavonoid content, and assumed that the main pigment was carotenoid, but did not conduct further investigations [27]. A systematic study of the color change phenomenon in *E. chrysantha* can provide a theoretical basis for genetic improvement and breeding of its flower color, as well as offer insights into flower color changes in other plants.

## Results

### Analysis of flower color phenotypic parameters and pigment content

**Fig. 1:**
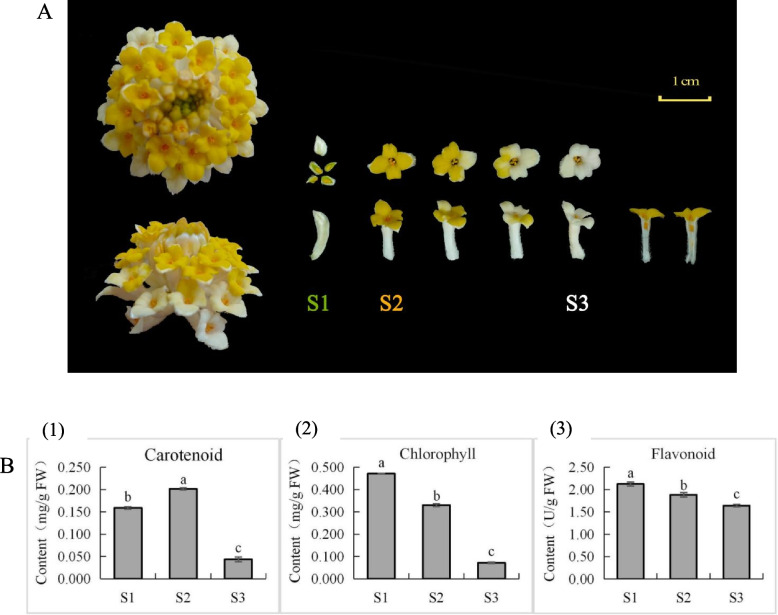
Color change and pigment content changes during *E. chrysantha* flowering process. **A ***E. chrysantha* color change process. S1: bud stage—green tepal; S2: full bloom flowering stage—yellow tepal; S3: late flowering stage—white tepal. **B ***E. chrysantha* pigment content changes during flowering. (1) Total carotenoid content at 3 stages. (2) Total chlorophyll content at 3 stages. (3) Total flavonoid content at 3 stages. Data in each period of the bar chart are presented as mean ± standard deviation. The lowercase letters on the bars represent significant differences (*P* < 0.05)

Significant variations are observed in the phenotypic parameters of color in *E. chrysantha* flowers throughout the color change process (Fig. 1A), as indicated by the data presented in Table 1. As the flowers mature, apart from the gradual increase in lightness (L* value), the hue values (a*) and (b*) as well as the chroma value (C) exhibit an initial ascent followed by a subsequent descent trend.

**Table 1:** Chromaticity values at different stages

Stage	Color	L*	a*	b*	C
S1	Green	80.94 ± 0.36 c	-23.96 ± 0.28 c	47.11 ± 0.41 b	52.85 ± 0.48 b
S2	Yellow	84.43 ± 0.30 b	7.05 ± 0.12 a	89.04 ± 0.19 a	89.32 ± 0.19 a
S3	White	98.57 ± 0.24 a	2.41 ± 0.34 b	8.51 ± 0.28 c	8.85 ± 0.35 c

The asterisk (*) in L*, a*, and b* is part of the name. The data in the table are mean ± standard deviation, and the lowercase letters marked after the data indicate significant difference (*P* < 0.05). The same applies below

The content of carotenoids, chlorophyll, and flavonoids in the floral petals also exhibited significant differences (Fig. 1B). Carotenoid content exhibited an initial increase followed by a sharp decrease, whereas both chlorophyll and flavonoid levels showed a declining trend as the flower developed. Notably, the decline in chlorophyll content was more pronounced.

Through correlation analysis between color indices and pigment contents during three stages of *E. chrysantha* flower color transformation (Table 2), significant relationships were observed. All pigment contents exhibited significant negative correlations with lightness (L* value) at a highly significant level (*P* < 0.01), indicating that lower pigment contents were associated with lighter flower colors. Moreover, the carotenoid content showed highly significant positive correlations with hue (b* value) and chroma (C value) (*P* < 0.01), highlighting the pronounced relationship between carotenoid content in *E. chrysantha* tepals and both the yellow color attribute and color vividness, emphasizing the pivotal role of carotenoids as key pigments in the coloration process. Notably, the chlorophyll content displayed a significant negative correlation with hue (a* value) (*P* < 0.05), while exhibiting a significant positive correlation with chroma (C value) (*P* < 0.05), suggesting a certain association between chlorophyll content and the green color attribute and color vividness of the flower. These findings underscore the critical impact of carotenoid and chlorophyll content on *E. chrysantha* flower coloration.

**Table 2:** Correlation matrix between chromaticity values and pigment content

Index	L*	a*	b*	*C*
Carotenoid content	-0.899^a^	-1.21	0.959^a^	0.978^a^
Chlorophyll content	-0.984^a^	-0.674^b^	0.618	0.677^b^
Flavonoid content	-0.938^a^	-0.780^b^	0.478	0.544

The asterisk (*) in L*, a*, and b* is part of the name. Pearson correlation analysis was used; ^a^indicates a significant correlation at the 0.01 level, and ^b^indicates a significant correlation at the 0.05 level

### Analysis of carotenoid metabolites

**Fig. 2:**
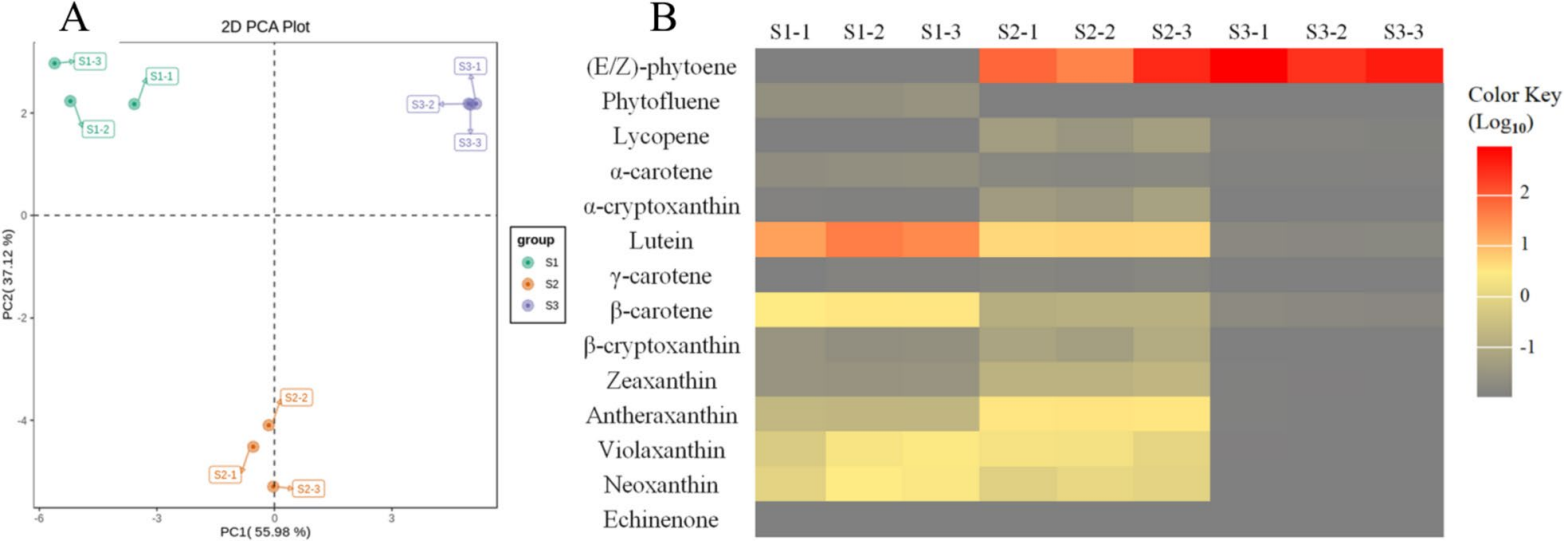
Preliminary analysis of metabolome data. **A** PCA score plot of mass spectrometry data for each sample. The sampling groups are color coded as follows: green = S1; orange = S2; purple = S3. **B** Heatmap of DAMs’ contents in carotenoid biosynthesis pathway at 3 stages. The horizontal axis represents nine sets of experimental samples with three biological replicates for each of the three periods, and the vertical axis represents 14 metabolites annotated to ko00906. The more gray the color, the lower the content; the more red the color, the higher the content

A total of 68 metabolites were examined in this study, and 33 substances were detected among the nine *E. chrysantha* samples collected at three distinct time points (see Table S1 for the detection results). Principal component analysis was conducted on the metabolite data obtained from these samples, and the score plot is presented in Fig. 2A. The plot revealed significant inter-group differences in the composition of carotenoid metabolites within the *E. chrysantha* samples during the process of flower color change. Conversely, within each time point, the samples exhibited relatively stable contents with minor fluctuations, indicating good biological reproducibility and suitability for subsequent analyses.

We focused on the annotation of 14 metabolites associated with the carotenoid biosynthesis pathway (ko00906), as illustrated in Fig. 2B. Significant variations in both the types and contents of carotenoids were observed in the tepal samples of *E. chrysantha* across different time points. In S1, a total of 12 carotenoids were detected, with a combined content of 95.859 μg/g. The major constituents included lutein, β-carotene, zeaxanthin, neoxanthin, and antheraxanthin. In S2, a total of 13 carotenoids were detected, with a combined content of 155.933 μg/g. The major constituents were phytoene, lutein, antheraxanthin, zeaxanthin, and neoxanthin. In S3, a total of 9 carotenoids were detected, with a cumulative content of 155.338 μg/g. The predominant compound was phytoene.

During the flower development process, different types of carotenoids exhibited distinct patterns of change across the three time periods. From S1 to S2, significant variations were observed in 10 metabolites, with most showing an increasing trend. For instance, phytoene, which was undetectable in S1, reached a high level of 107.550 μg/g in S2. Similarly, lycopene emerged in S2 at 1.359 μg/g. Antheraxanthin increased from 2.670 μg/g to 9.726 μg/g, representing a 3.64-fold increase. α-cryptoxanthin rose from 0.083 μg/g to 1.372 μg/g, marking a substantial 16.52-fold increase. Additionally, there was a roughly 2.4-fold increase observed in zeaxanthin (from 0.989 μg/g to 2.493 μg/g), β-cryptoxanthin (from 0.840 μg/g to 1.793 μg/g), and γ-carotene (from 0.121 μg/g to 0.295 μg/g). In contrast, lutein and β-carotene exhibited significant decreases, with contents in S2 being less than 30% of those in S1. Specifically, lutein decreased from 72.353 μg/g to 20.067 μg/g, and β-carotene decreased from 7.894 μg/g to 2.295 μg/g. Additionally, the trace amounts of phytofluene detected in S1 were no longer detected in S2. The remaining four metabolites showed no significant changes, with neoxanthin and zeaxanthin maintaining levels between 4–5 μg/g, and a slight decrease observed in α-carotene and echinenone. From S2 to S3, the flower color transitioned from yellow to white. Apart from a slight increase in phytoene from 107.550 μg/g to 154.070 μg/g, all other detected metabolites exhibited a clear decreasing trend, some even reaching undetectable levels. A comparative analysis between S1 and S3 revealed that, except for phytoene and lycopene, the remaining 12 carotenoid metabolites were significantly lower in S3 compared to S1.

Based on the findings, it can be postulated that the elevated levels of lutein and β-carotene in S1 are likely the primary contributors to the yellow color attribute of the floral tepals during the bud stage. In S2, both the diversity and content of carotenoids reached their peak among the three time periods. The notable increments in antheraxanthin, zeaxanthin, lycopene, and α-cryptoxanthin significantly enhanced the yellow color attribute and vividness of the tepals. In S3, except for the colorless phytoene, all other carotenoid compounds experienced a significant reduction to remarkably low levels. This decline in carotenoid content constitutes a pivotal factor underlying the manifestation of the pale white color of the tepals during this stage.

### Analysis of transcriptomic data

To investigate the underlying mechanisms contributing to the color-transition observed in *E. chrysantha* across three different developmental stages, transcriptomic sequencing analysis was performed to examine the expression profiles of genes associated with metabolite regulation. A total of 9 samples were sequenced using the Illumina NovaSeq 6000 platform, generating approximately 44.28 million raw reads. After quality control and data filtering, 41.32 million clean reads were obtained, ranging from 4.36 to 4.86 million clean reads per individual sample. The total sequencing data output for all samples reached 61.97 G, with each sample having clean bases exceeding 6.5 G. The error rate was controlled within 0.03%. Detailed information on sequencing output and quality statistics can be found in Table S2.

As *E. chrysantha* lacks a reference genome sequence, the transcript sequences assembled by Trinity were utilized as the reference sequences for subsequent analysis. After performing hierarchical clustering using Corset, the longest sequences from each cluster were selected as Unigenes for further analysis. A total of 153,301 Unigenes were obtained (as shown in Table S3). To annotate these Unigenes, sequence comparisons were conducted against the KEGG, NR, Swiss-Prot, GO, KOG, Trembl, and Pfam databases. As a result, 75,447 Unigenes (49.21% of the total) obtained annotation information (Table S4). For the high-quality clean reads obtained after filtering the sequencing data from the nine samples, mapping was performed against the reference sequences derived from transcriptome assembly. The number of mapped reads per sample accounted for 76.97% to 82.83% of the total filtered data, as shown in Table S5.

The evaluation of experimental reproducibility and the reliability of differentially expressed genes (DEGs) was performed based on the Pearson correlation coefficient (R^2^). The correlation analysis of the samples in this study (as shown in Fig. S1) indicated good correlation among the samples within each group, confirming the repeatability of the experimental procedures.

The criteria used for selecting DEGs were |log_2_Fold Change|≥ 1 and FDR < 0.05. After applying these criteria, the total number of DEGs, upregulated genes, and downregulated genes were calculated for each comparison, as depicted in Fig. S2. Furthermore, Venn diagrams were constructed based on the pairwise comparisons between different time periods, as shown in Fig. S3.

Upon annotating the DEGs from pairwise comparisons to the KEGG database [28, 29], the number of DEGs in each KEGG pathway was calculated. Significant enrichment analysis was conducted on a pathway-based level to identify pathways that were significantly enriched in DEGs compared to the entire genome background. The top 20 significantly enriched KEGG pathways were listed in Table S6. The analysis revealed that the carotenoid biosynthesis pathway (ko00906) displayed the most pronounced differences during the transition from S1 to S2. Notably, this pathway remained among the top pathways exhibiting significant changes in both the S2 to S3 and S1 to S3 comparisons, thereby providing further evidence for the pivotal role of carotenoids as crucial pigments driving the color variation in *E. chrysantha* flowers. Additionally, the biosynthesis of secondary metabolites pathway (ko01110) was consistently observed across all comparison groups. Furthermore, the plant hormone signal transduction pathway (ko04075) emerged as the most significantly enriched pathway in the S2 vs. S3 and S1 vs. S3 comparisons, indicating its critical involvement in the process of *E. chrysantha* flower color change.

### DEGs in the carotenoid biosynthesis pathway

Based on the KEGG annotation results and comprehensive comparison with various databases, a total of 134 DEGs were annotated to the carotenoid biosynthesis pathway (ko00906) in the S1 vs S2, S2 vs S3, and S1 vs S3 comparisons, with 80, 85, and 100 genes respectively. After filtering out genes with low expression abundance (FPKM < 50) and those located on peripheral pathway branches, 33 genes were further identified (Table S7), including 4 phytoene synthase genes (*PSY*), 5 15-cis-phytoene desaturase genes (*PDS*), 2 zeta-carotene desaturase genes (*ZDS*), 1 zeta-carotene isomerase gene (*Z-ISO*), 4 prolycopene isomerase genes (*CRTISO*), 1 beta-carotene isomerase gene (*DWARF27*), 3 beta-carotene 3-hydroxylase genes (*crtZ*), 2 zeaxanthin epoxidase genes (*ZEP*), 1 capsanthin/capsorubin synthase gene (*CCS1*), 8 9-cis-epoxycarotenoid dioxygenase genes (*NCED*), 1 xanthoxin dehydrogenase gene (*ABA2*), and 1 beta-ring hydroxylase gene (*CYP97A3*).

**Fig. 3:**
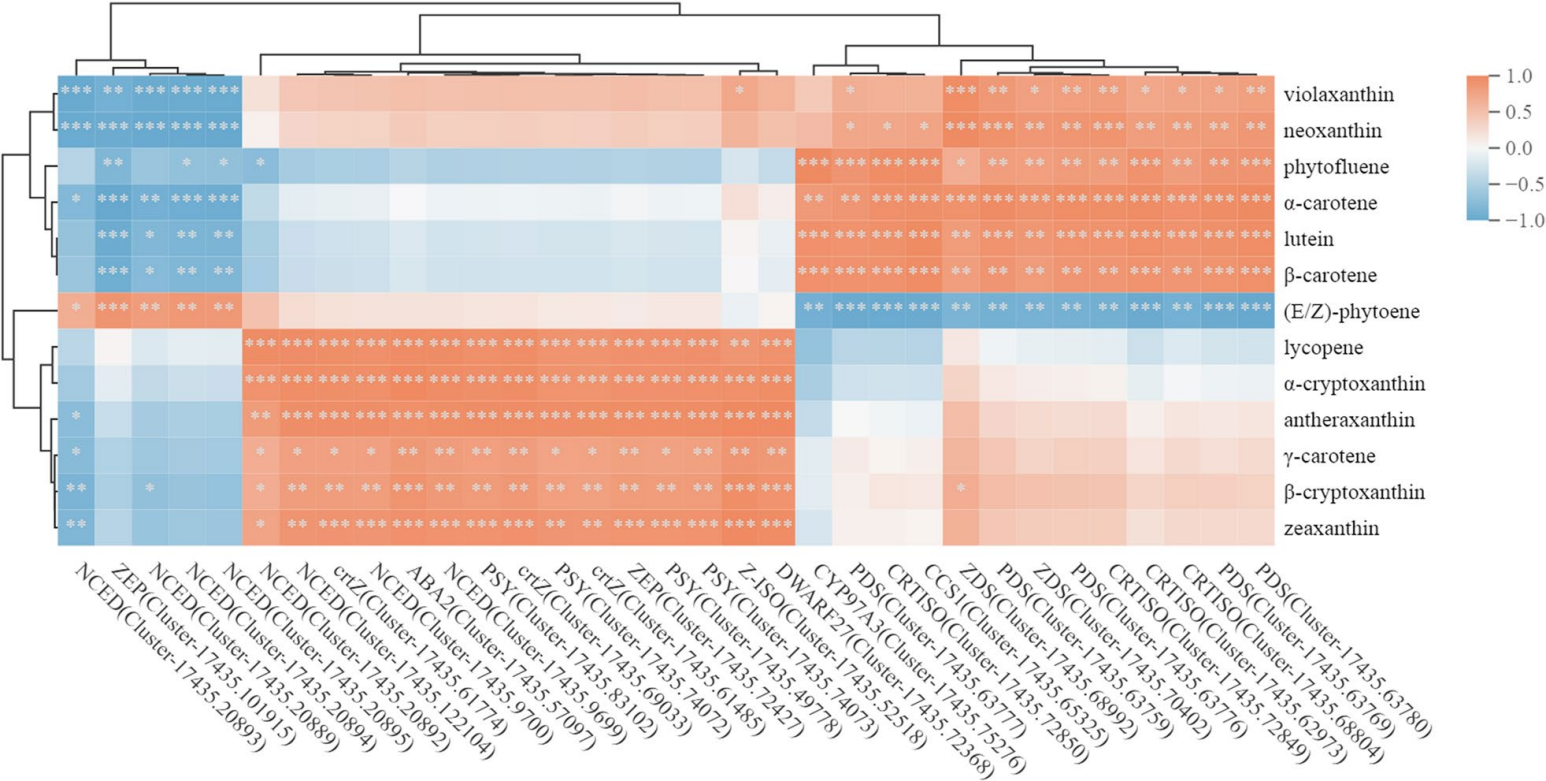
Correlation clustering heatmap of DEGs and DAMs related to carotenoid biosynthesis. Red color represents positive correlation, blue color represents negative correlation, and the intensity of the color indicates the strength of the correlation; *** indicates *P* < 0.001, ** indicates *P* < 0.01, and * indicates *P* < 0.05

To validate the reliability of the transcriptome data, ten genes were selected for qRT-PCR validation based on their high FPKM values, significant differential expression, and their presumed importance in coloration. The selected genes included 2 *PSY* (Cluster-17435.74072, Cluster-17435.83102), *NCED* (Cluster-17435.122104, Cluster-17435.20894), 1 *PDS* (Cluster-17435.63780), *ZDS* (Cluster-17435.68992), *Z-ISO* (Cluster-17435.52518), *crtZ* (Cluster-17435.61774), *ZEP* (Cluster-17435.72427), and *SGR* (Cluster-17435.63413). The primer sequences used for qRT-PCR can be found in Table S8, and the results of the qRT-PCR analysis are provided in Table S9. Linear regression analysis demonstrated a good fit between the Log_2_(FPKM) values obtained from the transcriptome data and the Log_2_(2^−ΔΔCt^) values obtained from the qRT-PCR data, with an R^2^ value of 0.8045 (Fig. S4). The quantitative validation results of the ten genes were in substantial agreement with the expression trends observed in the transcriptome sequencing data. This indicates a strong correlation between the two datasets, validating the reliability of the transcriptome analysis. Further, to validate the regulatory impact of the 33 selected genes on the metabolites, a correlation clustering analysis was conducted between the 33 DEGs and 13 differentially accumulating metabolites (DAMs) (excluding echinenone for its extreme low content) in the carotenoid biosynthesis pathway. The results, as shown in Fig. 3, revealed a close correlation between each gene and multiple metabolites. Specifically, the synthetic genes showed a positive correlation with the metabolite levels, while the degradation genes exhibited a negative correlation with the metabolite levels.

**Fig. 4:**
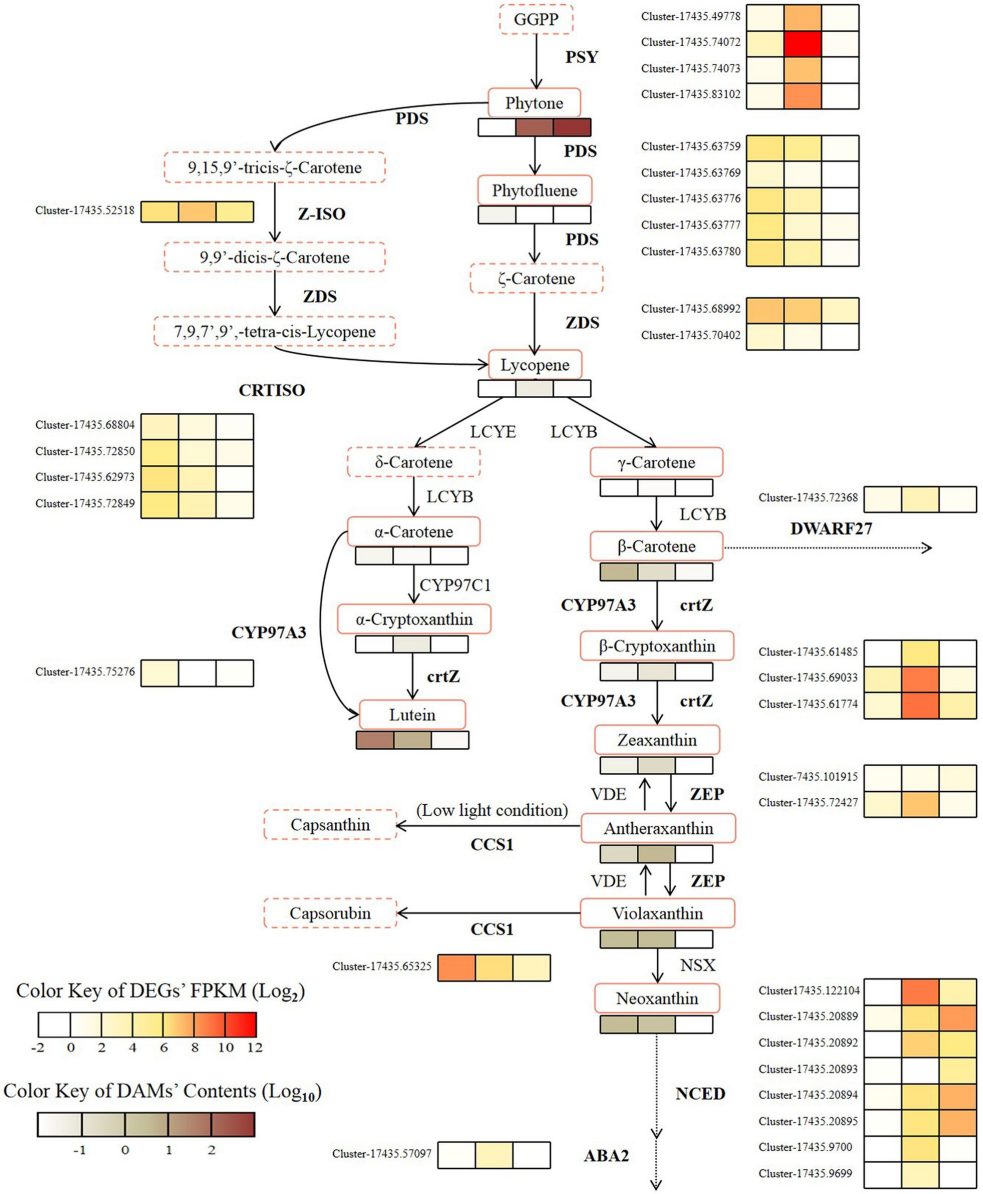
Diagram of selected DEGs along the carotenoid biosynthesis pathway. The three boxes next to the gene and below the metabolite are labelled S1, S2, and S3 from left to right, the same below

The selected 33 genes showed distinct expression patterns across the three measured periods. Given the upstream and downstream relationships of the enzymes and metabolites controlled by these genes, and because some enzymes are located at multiple branches of the pathway, a comprehensive analysis integrating the metabolomic data was conducted to accurately assess the regulatory impact of each gene on the metabolites. Based on the KEGG database and the metabolomic data, a heatmap was generated to visualize the differential expression of DEGs along the pathway at different time points (Fig. 4).

The heatmap analysis reveals that the majority of genes associated with synthesis display their highest expression levels in either S1 or S2. Genes such as *PSY*, *Z-ISO*, *crtZ*, and *ZEP* exhibit an ascending followed by a descending expression trend, with S2 showing the most prominent expression. This expression pattern aligns with the changes in the yellow color attribute and color vividness of the tepals. Although *PDS* and *ZDS* continuously decrease, several genes within these 2 groups exhibit strong expression in S1, indicating their significant role in metabolite synthesis and accumulation. On the other hand, genes related to degradation begin to exhibit elevated expression in S2 and reach their peak in S3. Among them, it is inferred that *NCED* plays a key role in degradation based on the number and FPKM levels of homologous genes. The combined accumulation of S1 (high synthesis > low degradation) and S2 (high synthesis > high degradation) leads to the highest overall content of key metabolites in S2. The expression intensity of degradation-related genes in S3 is significantly higher than that of synthesis-related genes (high degradation >  > low synthesis), resulting in the continuous degradation of metabolites and a lighter flower color. These findings highlight the coordinated regulation of carotenoid content in the tepals by both carotenoid synthesis enzymes and degradation enzymes.

### DEGs in the porphyrin and chlorophyll metabolism pathway

Based on the KEGG annotation and enrichment results, the comparison between S1 and S3 revealed a significant enrichment of the porphyrin and chlorophyll metabolism pathway (ko00860). This finding is particularly noteworthy considering the observed greenish color of the *E. chrysantha* tepals in S1, accompanied by a higher content of chlorophyll. These results suggest a potential influence of chlorophyll on tepal pigmentation. Consequently, a detailed analysis of the pathways associated with chlorophyll metabolism was performed.

**Fig. 5:**
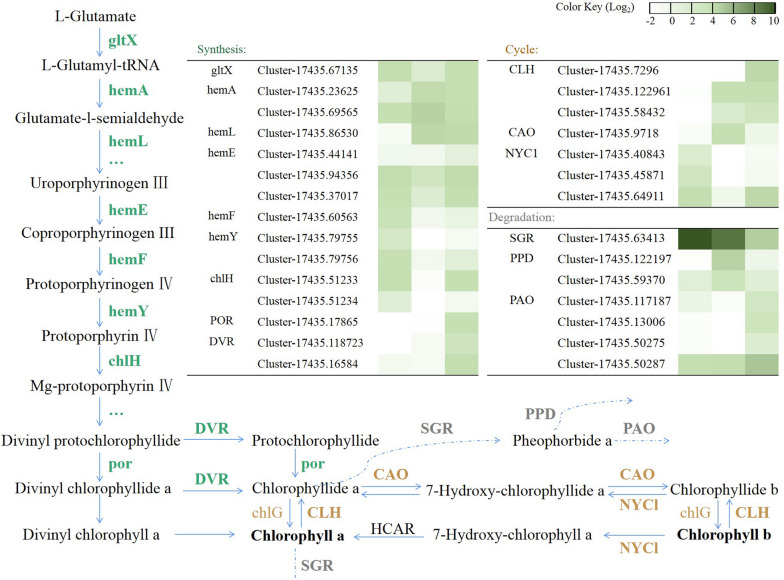
Expression profile of selected DEGs along porphyrin and chlorophyll metabolism pathway. Upper right: FPKM value heatmap of DEGs in the porphyrin and chlorophyll metabolism pathway. Outer periphery: Main processes regulated by DEGs participating

In the porphyrin and chlorophyll metabolism pathway, a total of 103 DEGs were identified in the S1 vs S2, S2 vs S3, and S1 vs S3 comparisons, with 59, 65, and 73 genes respectively. These DEGs were further screened, focusing on genes located in the main pathway and exhibiting high expression levels (FPKM > 10). Consequently, a subset of 29 genes was selected and a heatmap was generated based on their FPKM values, as depicted in Fig. 5.

From the heatmap, it is evident that the overall expression intensity of DEGs in the degradation phase is the highest among the three stages. *SGR* (Cluster-17435.63413) emerges as the most prominently expressed and highest peak FPKM gene in this pathway, with expression levels of 1871.69, 1463.97, and 277.41 in each respective stage. Similarly, *PPD* (Cluster-17435.122197) and *PAO* (Cluster-17435.50287) reach FPKM values of 200.39 and 392.97 in S2 and S3, surpassing the highest expression levels of DEGs in other stages.

It is speculated that the green color of the flower tepals in S1 may be attributed to the accumulation of chlorophyll, which is regulated by the porphyrin and chlorophyll metabolism pathway prior to the examined stages. During the examined stages, the gene expression levels in the tepals of *E. chrysantha* indicate a deceleration in synthesis and an enhancement in degradation in S1. Subsequently, in S2 and S3, degradation surpasses synthesis, leading to the continuous depletion and reduction of chlorophyll. Considering the position within the pathway and the gene expression intensity, *SGR* is likely a key gene involved in chlorophyll degradation.

### Analysis of transcription factors

The prediction of transcription factors (TFs) and transcriptional regulators (TRs) was conducted using the iTAK software. A total of 2,740 TFs from 67 families and 1,034 TRs from 25 families were predicted in the experiment. The top ten families with the highest number of members were C2H2 (188 TFs), C3H (188 TFs), bHLH (177 TFs), MYB-related (171 TFs), Unclassified (157 TRs), AP2/ERF-ERF (139 TFs), NAC (136 TFs), WRKY (132 TFs), SNF2 (122 TRs), and MYB (120 TFs).

Based on the results of GO functional annotation, a total of 13 differentially expressed transcription factors (TFs) were identified and found to be involved in carotenoid metabolic process (GO:0016116) and carotenoid biosynthetic process (GO:0016117) in the S1 vs S2, S2 vs S3, and S1 vs S3 comparisons. These TFs predominantly belonged to the C2H2, MYB-related, and SET families, as summarized in Table 3. Among them, based on the expression levels and fold change, it is speculated that two C2H2 transcription factors (Cluster-17435.62534 and Cluster-17435.62989) and one MYB transcription factor (Cluster-17435.49372) may play significant roles in the regulation of carotenoid metabolism.

**Table 3:** Differentially expressed TFs and TRs related to carotenoid biosynthesis in 3 comparison groups

A vs B	ID	Family	A-FPKM	B-FPKM	FC	Type
S1 vs S2	Cluster-17435.9321	MYB-related	0.55	16.98	30.87	up
	Cluster-17435.63285	C2H2	0	12.07	Inf	up
	Cluster-17435.54658	SET	4.81	6.54	1.36	up
	Cluster-17435.54660	SET	4.63	6.19	1.34	up
	Cluster-17435.54656	SET	0.22	1.51	6.86	up
	Cluster-17435.69830	B3	3.55	0.33	0.09	down
S2 vs S3	Cluster-17435.62534	C2H2	5539.50	355.97	0.06	down
	Cluster-17435.49372	MYB	6.65	34.04	5.12	up
	Cluster-17435.62989	C2H2	31.58	9.70	0.31	down
	Cluster-17435.11349	MYB-related	0.92	0.50	0.54	down
	Cluster-17435.9321	MYB-related	16.98	0	0	down
	Cluster-17435.63285	C2H2	12.07	0	0	down
	Cluster-17435.119353	MYB-related	1.98	0	0	down
	Cluster-17435.69831	B3	0.61	0	0	down
S1 vs S3	Cluster-17435.62534	C2H2	6530.26	355.97	0.05	down
	Cluster-17435.62989	C2H2	55.50	9.70	0.17	down
	Cluster-17435.63412	C2H2	9.38	2.60	0.28	down
	Cluster-17435.49372	MYB	9.13	34.04	3.73	up
	Cluster-17435.54656	SET	0.22	1.30	5.91	up

To further verify the regulatory impact of the 3 selected TFs on the related genes and metabolites, a Pearson correlation analysis was performed between the three TFs and the previously analyzed 13 carotenoid metabolites and 33 DEGs. A correlation threshold of 0.8 with a significance level of *P* < 0.05 was set, and the correlation results are depicted in Fig. S5. From the correlation network plot, it can be seen that the three transcription factors obtained through annotation and screening analysis show close correlations with carotenoid metabolites and key genes. In addition, it was found that two transcription factors in the C2H2 family (Cluster-17435.62534 and Cluster-17435.62989) are positively correlated with the content of various metabolites and the expression of most synthetic genes, but negatively correlated with the expression of degradation genes; while the MYB transcription factor (Cluster-17435.49372) is negatively correlated with the content of various metabolites and the expression of most synthetic genes.

## Discussion

### Carotenoid metabolites and floral color-transition

Studies have shown that the changes in the levels of floral antheraxanthin, zeaxanthin, lycopene, and α-cryptoxanthin in the tepals of *E. chrysantha* during its three developmental stages closely align with the observed phenotypic variations in flower color. These metabolites exhibit significant variations in their content at different time points, suggesting their crucial role in petal pigmentation, with a possible emphasis on antheraxanthin. This finding shares certain similarities with previous investigations conducted by Guo et al*.* [30] on yellow coloration in *Brassica napus*, Xia et al*.* [31] on color transition in *Lonicera japonica* flower petals, Huang et al*.* [32] on color variation in *Dendrobium chrysotoxum* flowers, and Yamagishi et al. [33] on coloration in *Lilium brownii* var. *viridulum* flowers. Prior to the widespread application of metabolomic profiling techniques, research on carotenoids primarily focused on compounds such as lutein, zeaxanthin, lycopene, and β-carotene, while the study of antheraxanthin, particularly in floral organs, has been relatively limited. Thus, further studies are needed to expand our understanding of antheraxanthin and their contributions to flower coloration.

### DEGs underlying the color-transition of *E. chrysantha* petals

For the first time, this study applied transcriptome sequencing technology to *E. chrysantha*, constructing a transcriptome database of *E. chrysantha* tepals with high sequencing quality, providing reliable data support for subsequent research. Through assembly and splicing, we obtained 153,301 Unigenes, of which only 75,447 (49.21%) were annotated. Half of the sequences were not annotated, which may be attributed to two primary reasons: (1) the lack of reference genomes for *E. chrysantha* and its closely related species, as well as the limited availability of relevant reference information. Therefore, the transcripts of *E. chrysantha* can only be compared with public databases such as KEGG, GO, Swissprot, Nr, TeEMBL, KOG, and Pfam. However, the genomic differences between different species are substantial, which may result in low annotation rates; *E. chrysantha*, as a distinct species, may possess a significant number of unknown genes that differ from those of other species; (2) there may be certain discrepancies between the transcripts and their annotation obtained through a series of software processing and analysis, and the actual transcription situation in living tissue cells, including but not limited to incomplete sequences due to the instability and susceptibility to degradation of RNA, accuracy during assembly and splicing, and strict parameters set to avoid false positives during annotation.

Carotenoid accumulation in plant tissues is intricately linked to their synthesis, degradation, and storage processes. Any changes occurring in these pathways can potentially alter the levels of carotenoids and affect the phenotype. In the synthesis process, *PSY*, the gene commonly regarded as the rate-limiting enzyme, exhibits high expression levels in the transcriptome of *E. chrysantha* flowers, leading to the rapid accumulation of phytoene. This phenomenon is consistent with the experimental findings of Zhou et al*.* [34] and *Liu* [35]. The high expression of *crtZ* and *ZEP* genes may play a crucial role in the significant increase of antheraxanthin, particularly during the bloom flowering period. The specific effects of different genes need to be further elucidated through gene function validation experiments. Moreover, considering that antheraxanthin are involved in the reversible xanthophyll cycle, the regulatory network governing these processes is likely to be more intricate. During the degradation process, *NCED* exhibits relatively low expression levels in the early stages of development but gradually increases upon flower opening, reaching its highest expression levels during the late flowering period. This suggests a close correlation between *NCED* and carotenoid degradation, as well as the phenomenon of flower color shifting towards white. These findings align with the research conducted by Jia [36] on white-flowered *B. napus* and Huang et al. [32] on *D. chrysanthum*. Based on this speculation, it is inferred that interfering with the expression of the *NCED* genes in *E. chrysantha* may alter the color fading phenomenon of the tepals from S2 to S3 by reducing its degradation of carotenoid metabolites.

To investigate the reasons for the consistent decrease in chlorophyll content during three growth stages of *E. chrysantha*, DEGs analysis was conducted on the porphyrin and chlorophyll metabolism pathway. The analysis suggests that the high expression of *SGR* is closely correlated with the continuous degradation of chlorophyll, which is consistent with the research results of Tang et al. [37] on the chlorophyll degradation pathway. Consequently, *SGR* has an important impact on plant coloration, a phenomenon that had previously been demonstrated by Zhu et al. [38] in their study of the brown mutant of navel orange (*Citrus sinensis* ‘Zongcheng’): mutation of *SGR* led to the degradation of chlorophyll in the fruit peel being blocked, causing green chlorophyll to accumulate with orange, resulting in a brown phenotype in the fruit peel.

It is worth noting that in the transcriptome data of the three periods of flower color change, the KEGG pathway annotation and enrichment results of the DEGs showed that secondary metabolite biosynthesis (ko01110) appeared in all comparison groups, and the plant hormone signal transduction (ko04075) was the most significantly enriched pathway in both S2 vs S3 and S1 vs S3 comparisons, underscoring these two pathways’ critical role in the flower color change process of *E. chrysantha*. Upstream in the carotenoid metabolism pathway lies the MEP (methylerythritol phosphate) pathway, which not only synthesizes carotenoids but also produces various secondary metabolites such as chlorophylls, gibberellins, cytokinins, and terpenoids [39–42]. This pathway is an extensive network of secondary metabolism, encompassing the synthesis of multiple secondary metabolites. Carotenoids also serve as vital precursors for several plant hormones, including abscisic acid and strigolactones [43, 44]. Notably, the enzyme NCEDs plays a crucial role by catalyzing the breakdown of compounds like violaxanthin and neoxanthin, leading to the formation of precursor substances for abscisic acid. In this study, a significant number of *NCED* homologous genes were identified and annotated in *E. chrysantha*, and their overall expression intensity increased during flower development. Therefore, it is plausible that *NCED* not only contribute significantly to the degradation of various metabolites within the β-branch of carotenoid metabolism but also exhibit a strong association with the highly active hormone signal transduction pathways. Furthermore, the degradation of chlorophyll is positively correlated with ethylene signal transduction [45]. These findings collectively emphasize that flower development and maturation involve a complex interplay of pigment synthesis, degradation, and a range of intricate physiological processes.

### The intrinsic mechanism of flower color-transition in *E. chrysantha*

**Fig. 6:**
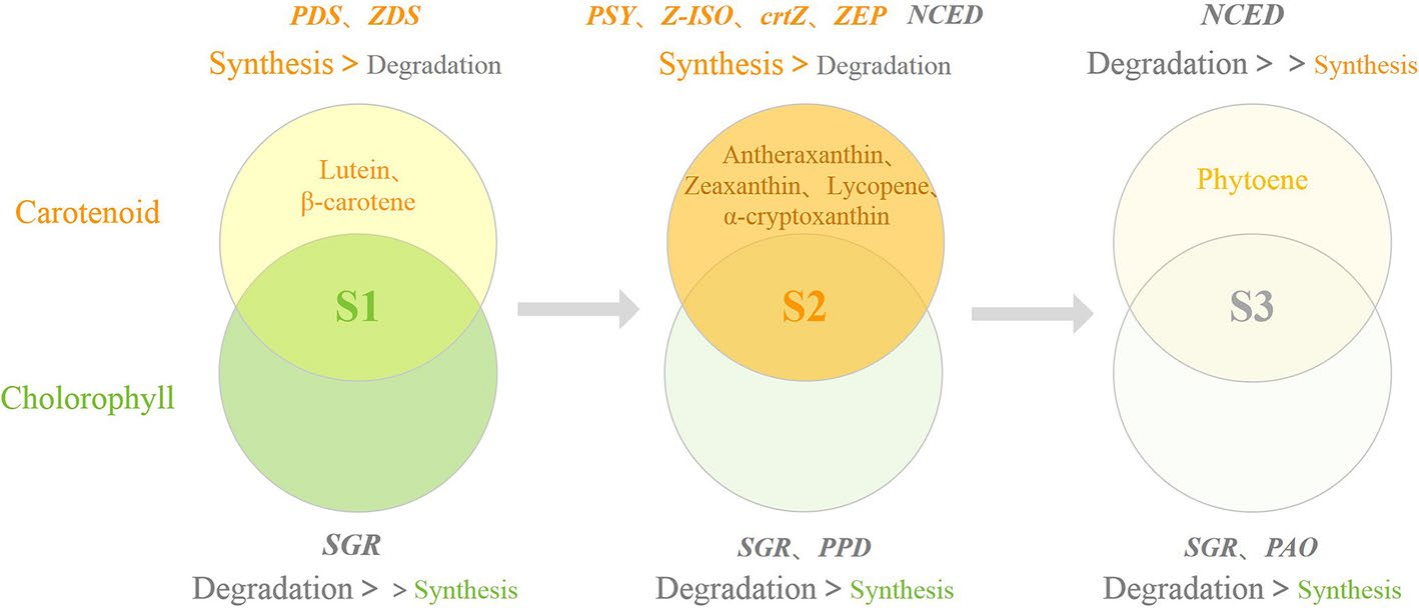
Schematic diagram of the mechanism of floral color change in *E. chrysantha* tepals. The shade of color of the circle indicates the amount of pigment content. Above: Changes in carotenoids across three time periods. Below: Changes in chlorophyll across three time periods. Inside the circle: Metabolites with significant impact on coloration. Outside the circle: Potential regulatory genes and main synthesis/degradation patterns

Based on the findings above, the mechanism underlying the color-transition in *E. chrysantha* tepals has been initially elucidated (Fig. 6), but the actual biological regulatory mechanisms involved are highly complex: after plant cells undergo transcription and expression, there are also processes such as post-transcriptional modifications, protein translation, and post-translational modifications that occur, and transcriptional regulation is ultimately reflected at the metabolic level; meanwhile, the regulatory mechanisms of enzymes within cells, such as substrate competition and cooperativity, are still poorly understood; additionally, it is unclear how potential transcription factors such as C2H2 and MYB function in this process. Thus, further investigation into the impact of internal and external factors is required to elucidate the biological mechanisms of *E. chrysantha* floral color changes, indicating that more rigorous research and validation efforts are indispensable.

## Conclusion

In this study, we focused on the tepals of *E. chrysantha* and examined the pronounced color-transition observed during its flowering process. Through meticulous measurements of phenotypic parameters at three distinct stages, we quantified the dynamic changes in flower color. Furthermore, we analyzed the pigment content at different developmental phases to unravel the accumulation patterns of each pigment during the blooming of *E. chrysantha*. By identifying the pivotal pigments responsible for petal coloration, we proceeded to perform comprehensive qualitative and quantitative analyses of these key metabolites, shedding light on their roles in the color-changing process. Additionally, we integrated transcriptome sequencing analysis, allowing us to pinpoint crucial regulatory genes and TFs, which were further validated through qRT-PCR experiments. Overall, our study presents a systematic investigation, spanning from phenotypic observations to molecular insights, that unravels the intrinsic regulatory mechanisms underlying flower color-transition in *E. chrysantha*. These findings not only contribute to a deeper understanding of the complex biological regulation of flower color but also provide a theoretical basis for future molecular-level manipulation of flower coloration and the breeding of novel varieties.

## Materials and methods

### Plant materials

The experiment was conducted using healthy *E. chrysantha* tepals, which were cultivated in a consistent habitat without any diseases or pests, within the premises of Central South University of Forestry and Technology. The sampling time for the materials is on the morning of March 8th, 2022. Considering the developmental stages and actual coloration of *E. chrysantha* tepals, we selected three distinct periods with significant differences in tepal appearance (Fig. 1A): bud stage—green phase (S1), full bloom flowering stage—yellow phase (S2), and late flowering stage—white phase (S3). Upon collection, the visible parts of the tepals were promptly excised on ice, rinsed with distilled water to remove surface moisture, and partially utilized for color difference and pigment content measurements. The remaining portions were placed in centrifuge tubes and rapidly frozen in liquid nitrogen, followed by storage at -80℃ in an ultra-low temperature freezer for subsequent metabolomic and transcriptomic sequencing analyses. Each experimental group consisted of three biological replicates.

### Measurement of phenotypic parameters

The color phenotypes of *E. chrysantha* tepal samples at different stages were measured using a color difference meter (CM-700d, Konica Minolta, Japan). The lightness value (L*), hue value (a* and b*) of the petal samples were obtained. L*, a*, and b* are the three coordinates in the CIE Lab color space, which together describe the appearance of a color. L* indicates lightness, ranging from black (0) to white (100); a* indicates the variation from red (positive a values) to green (negative a values); b* indicates the variation from yellow (positive b values) to blue (negative b values). The chroma (C) value was calculated using the formula C = (a*^2^ + b*^2^)^1/2^, which indicates the vividness of a color. Five repeated measurements were conducted for each sample, and the average values were calculated to represent the color phenotype measurements for each stage.

### Quantification of carotenoid, chlorophyll, and flavonoid content

The carotenoid and chlorophyll content in the tepals of *E. chrysantha* were determined using a plant carotenoid content detection kit (Beijing Solarbio Science & Technology Co., Ltd) following the manufacturer's instructions: weigh 0.1 g (accurate to 0.01 g) of *E. chrysantha* tepals, cut it into small pieces, place them in a mortar, add 1 mL of distilled water and 10 mg of reagent 1 (provided in the kit), grind the pieces thoroughly in the dark environment and transfer the mixture into a 10 mL test tube; extract the mixture with 80% acetone and make up the volume to 10 mL with the extractant; seal the test tube with a rubber stopper and wrap it with tin foil; extract for 3 h (flip and mix the solution twice during the extraction); transfer 1 mL of the upper layer extract into a 1 mL glass cuvette; use 80% acetone as the reference solution and measure the absorbance at 470 nm, 646 nm, and 663 nm, which are recorded as A470, A646, and A663, respectively. Calculate the chlorophyll a concentration (Ca), chlorophyll b concentration (Cb), total chlorophyll concentration (CT), and carotenoid concentration (Cc) according to the following formulas: Ca (mg/L) = 12.21 × A663-2.81 × A646; Cb (mg/L) = 20.13 × A646-5.03 × A663; CT (mg/L) = Ca + Cb; Cc (mg/L) = 1000 × A470-3.27 × Ca-104 × Cb)/229; calculate the content of carotenoids and chlorophylls using the following formula: Content (mg/g) = C × V × F ÷ W, where C is the concentration of carotenoids or chlorophylls, V is the volume of the extractant (0.01 L), F is the dilution factor (1), and W is the weight of the sample (0.1 g).

The flavonoid content was performed according to the methods described by Pirie [46]. Briefly, 0.1 g of fresh petal samples were weighed and placed in a test tube containing 5 mL of 1% hydrochloric acid methanol extraction solution. The test tube was tightly sealed with a rubber stopper and kept in a refrigerator at 4℃ for 24 h for extraction. The absorbance of the extraction solution at 325 nm was measured using a UV spectrophotometer, with 1% hydrochloric acid methanol extraction solution serving as the reference solution. The relative content of flavonoids was expressed as U = OD325/g FW (optical density at 325 nm per gram of fresh weight).

### Sample preparation for metabolite analysis

(1) Retrieve the samples from the -80℃ ultra-low temperature freezer and grind them into a fine powder using a ball mill (30 Hz, 1 min); (2) Weigh 50 mg of the ground sample and dissolve it in 0.5 mL of a solvent mixture containing n-hexane, acetone, and ethanol (1:1:1, v/v/v), with 0.01% BHT (g/mL) added; (3) Vortex the solution for 20 min at room temperature, then centrifuge it for 5 min at 4℃ at 12,000 r/min, and collect the supernatant; (4) Repeat steps 2 and 3 once, and combine the two supernatants; (5) Concentrate the pooled extract and redissolve it in 100 μL of a mixture of methanol and methyl tert-butyl ether (1:1, v/v). Then filter the solution through a 0.22 μm filter and store in a brown sample bottle for further LC–MS/MS analysis.

### Chromatography-mass spectrometry conditions

The data acquisition system mainly comprises ultra-high performance liquid chromatography (UPLC) (ExionLC™ AD) and tandem mass spectrometry (MS/MS) (QTRAP®6500 +).

Chromatography conditions: (1) Chromatographic column: YMC C30 (3 μm, 100 mm × 2.0 mm i.d.); (2) Mobile phase composed of Phase A (methanol/acetonitrile 1:3, v/v with 0.01% BHT and 0.1% formic acid) and Phase B (methyl tert-butyl ether with 0.01% BHT); (3) Gradient elution program: initial condition—A/B 100:0 (V/V), hold for 3 min, then A/B remains at 100:0 (V/V), continue for 5 min, then A/B changes to 30:70 (V/V), hold for 9 min, then A/B changes to 5:95 (V/V), hold for 10 min, then A/B returns to initial condition—100:0 (V/V), hold for 11 min; (4) Flow rate is set at 0.8 mL/min; Column temperature maintained at 28℃; Sample size is 2 μL.

Mass spectrometry conditions: APCI source temperature maintained at 350℃, Curtain gas (CUR) pressure set at 25 psi. For each ion pair in Q-Trap 6500 + , the declustering potential (DP) and collision energy (CE) are optimized for scanning and detection.

### UPLC conditions

The sample extracts were analyzed using a UPLC-APCI-MS/MS system (UPLC, ExionLC™ AD; MS, Applied Biosystems 6500 Triple Quadrupole). The system was configured with an analytical column (YMC C30, 3 μm, 100 mm × 2.0 mm i.d), a solvent system (methanol/acetonitrile, 1:3, v/v, with 0.01% BHT and 0.1% formic acid in Solvent A, and methyl tert-butyl ether with 0.01% BHT in Solvent B), and a gradient elution program (start at 0% B for 0–3 min, increased to 70% B for 3–5 min, then increased to 95% B for 5–9 min, and finaly ramped back to 0% B for 10–11 min). The flow rate was set at 0.8 mL/min, the temperature maintained at 28 °C, and the injection volume was set at 2 μL.

### APCI-MS/MS conditions

The QTRAP® 6500 + LC–MS/MS System, a triple quadrupole-linear ion trap mass spectrometer equipped with an APCI heated nebulizer, was used to acquire both linear ion trap (LIT) and triple quadrupole (QQQ) scans. The instrument was operated in positive ion mode under the control of Analyst 1.6.3 software (Sciex). The APCI source parameters were carefully optimized as follows: ion source set to APCI + , source temperature maintained at 350 °C, and curtain gas kept at 25.0 psi. To ensure precise analysis of carotenoids, we employed scheduled multiple reaction monitoring (MRM) as a detection method. All data acquisitions were carried out using the Analyst 1.6.3 software. Multiquant 3.0.3 software was used to quantify all metabolites. To achieve the most effective analysis, we optimized both the declustering potentials (DP) and collision energies (CE) for each MRM transition. A specific set of MRM transitions were then monitored for each period, according to the elution of metabolites within the period.

### RNA quantification and qualification

The quality of RNA was assessed by monitoring degradation and contamination through the use of 1% agarose gels. Additionally, the purity of RNA was verified using the NanoPhotometer® spectrophotometer (IMPLEN, CA, USA). The concentration of RNA was measured using the Qubit® RNA Assay Kit in the Qubit® 2.0 Flurometer (Life Technologies, CA, USA). To evaluate the integrity of the RNA, the RNA Nano 6000 Assay Kit of the Bioanalyzer 2100 system (Agilent Technologies, CA, USA) was employed. The mRNA was then purified from the total RNA using poly-T oligo-attached magnetic beads. Fragmentation was achieved using divalent cations under elevated temperature in NEBNext First Strand Synthesis Reaction Buffer (5X). First strand cDNA synthesis was carried out using a random hexamer primer and M-MuLV Reverse Transcriptase (RNase H-). Subsequently, second strand cDNA synthesis was performed using DNA Polymerase I and RNase H. The remaining overhangs were then converted into blunt ends through exonuclease/polymerase activities. Following the adenylation of 3’ ends of the DNA fragments, NEBNext Adaptors with a hairpin loop structure were ligated in preparation for hybridization. To ensure selection of cDNA fragments precisely 250 ~ 300 bp in length, we employed the AMPure XP system (Beckman Coulter, Beverly, USA) to purify the library fragments. Then, we added 3 µl of USER Enzyme (NEB,USA) to the size-selected, adaptor-ligated cDNA for a 15 min incubation at 37℃, followed by a 5 min incubation at 95℃. Before PCR, we used Phusion High-Fidelity DNA polymerase, Universal PCR primers and Index (X) Primer for PCR amplification. Finally, we cleaned the PCR products using the AMPure XP system and evaluated the quality of the library on the Agilent Bioanalyzer 2100 system.

### Library preparation for transcriptome sequencing

For each sample, 1 µg of RNA was used as input material for the RNA sample preparations. Sequencing libraries were created using the NEBNext® UltraTM RNA Library Prep Kit for Illumina® (NEB, USA). To enable the assignment of sequences to each sample, index codes were added.

### Clustering and sequencing

The index-coded samples were clustered on a cBot Cluster Generation System using TruSeq PE Cluster Kit v3-cBot-HS (Illumia). Subsequently, the library preparations were sequenced on the Illumina Novaseq6000 platform, generating 150 bp paired-end reads.

### qRT-PCR analysis

Based on the transcriptome data results, 10 DEGs were selected for validation using real-time fluorescence quantitative PCR, with *Actin* serving as the reference gene. Each sample was analyzed with three replicates. Primer sequences for the reference gene and candidate genes were designed using the NCBI online primer design software, Primer-Blast, as shown in Table S8. Total RNA, which passed the quality assessment, was reverse transcribed into cDNA following the instructions of the MonScript™ RTIII All-in-One Mix with dsDNase Reverse Transcription Kit (Monad). Subsequently, the QuantiNova SYBR Green PCR Kit (QIAGEN) was used for real-time fluorescence quantitative PCR on the ABI7500 real-time PCR system. The relative gene expression levels were calculated using the 2^−ΔΔCt^ method.

### Supplementary Information

**Additional file 1:** **Table S1.** Test results of carotenoid metabolites.

**Additional file 2:** **Table S2.** Statistics on the output and quality.

**Additional file 3:** **Table S3.** Statistical table of assembly results.

**Additional file 4:** **Table S4.** Statistical table of annotation results.

**Additional file 5:** **Table S5.** Statistical table of camparison results.

**Additional file 6:** **Table S6.** Significantly enriched KEGG pathways of DEGs in 3 comparison groups.

**Additional file 7:** **Table S7.** DEGs involved in carotenoid biosynthesis.

**Additional file 8:** **Table S8.** Primer sequences.

**Additional file 9:** **Table S9.** qRT-PCR results.

**Additional file 10:** **Fig. S1.** Correlation heat map of mass spectrometry data for each sample. The horizontal and vertical axes represent nine samples with three biological replicates for each of the three periods. The color at the intersection represents the correlation between the two sets of samples, with redder colors indicating higher correlation and yellower colors indicating lower correlation. **Fig. S2.** Comparison of the number of DEGs between two stages. Two different stages of development with total differentially expressed genes (black), up-regulated genes (light gray), and down-regulated genes (dark gray). **Fig. S3.** Venn map of DEGs among different stages. **Fig. S4.** Validation analysis of transcriptome sequencing data. **Fig. S5.** Correlation network diagram of TFs with DAMs and DEGs. **A** Correlation between TFs and DAMs. **B** Correlation between TFs and DEGs. The circular shapes represent TFs, square shapes represent DAMs/DEGs; Red lines indicate positive correlations, while blue lines indicate negative correlations.

## Data Availability

All the raw RNA sequencing data has been submitted to NCBI Sequence Read Archive (SRA) database under the accession number PRJNA986669, https://www.ncbi.nlm.nih.gov/sra/PRJNA986669.

## Abbreviations

PSY: Phytoene synthase
PDS: 15-cis-phytoene desaturase
ZDS: Zeta-carotene desaturase
Z-ISO: Zeta-carotene isomerase
CRTISO: Prolycopene isomerase
CYP97A3: Beta-ring hydroxylase
crtZ: Beta-carotene 3-hydroxylase
ZEP: Zeaxanthin epoxidase
CCS1: Capsanthin/capsorubin synthase
DWARF27: Beta-carotene isomerase
NCED: 9-Cis-epoxycarotenoid dioxygenase
ABA2: Xanthoxin dehydrogenase
gltX: Glutamyl-tRNA synthetase
hemA: Glutamyl-tRNA reductase
hemL: Glutamate-1-semialdehyde 2,1-aminomutase
hemE: Uroporphyrinogen decarboxylase
hemF: Coproporphyrinogen III oxidase
hemY: Protoporphyrinogen/coproporphyrinogen III oxidase
chlH: Magnesium chelatase subunit H
POR: Protochlorophyllide reductase
DVR: Divinyl chlorophyllide a 8-vinyl-reductase
CLH: Chlorophyllase
CAO: Chlorophyllide a oxygenase
NYC1: Chlorophyll(ide) b reductase
SGR: Magnesium dechelatase
PAO: Pheophorbide a oxygenase
PPD: Pheophorbidase
