# Supplementary material for: Integrated transcriptome and metabolome analysis unveils the mechanism of color-transition in Edgeworthia chrysantha tepals
Source: BMC Plant Biol. 2023 Nov 16;23:567. doi: 10.1186/s12870-023-04585-1 (PMC10652483; doi:10.1186/s12870-023-04585-1)
Supplement: Supplementary file 10 — Additional file 10: Fig. S1. Correlation heat map of mass spectrometry data for each sample. The horizontal and vertical axes represent nine samples with three biological replicates for each of the three periods. The color at the intersection represents the correlation between the two sets of samples, with redder colors indicating higher correlation and yellower colors indicating lower correlation. Fig. S2. Comparison of the number of DEGs between two stages. Two different stages of development with total differentially expressed genes (black), up-regulated genes (light gray), and down-regulated genes (dark gray). Fig. S3. Venn map of DEGs among different stages. Fig. S4. Validation analysis of transcriptome sequencing data. Fig. S5. Correlation network diagram of TFs with DAMs and DEGs. A Correlation between TFs and DAMs. B Correlation between TFs and DEGs. The circular shapes represent TFs, square shapes represent DAMs/DEGs; Red lines indicate positive correlations, while blue lines indicate negative correlations. [file 12870_2023_4585_MOESM10_ESM.docx]

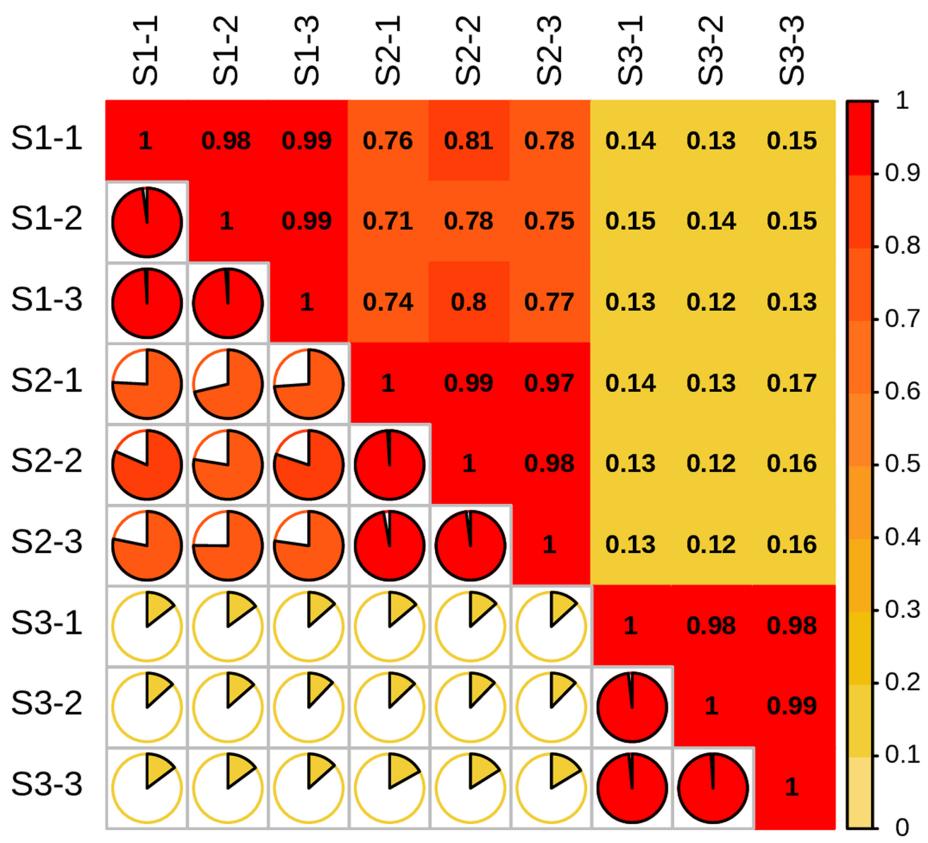


**Fig. S1** Correlation heat map of mass spectrometry data for each sample. The horizontal and vertical axes represent nine samples with three biological replicates for each of the three periods. The color at the intersection represents the correlation between the two sets of samples, with redder colors indicating higher correlation and yellower colors indicating lower correlation.


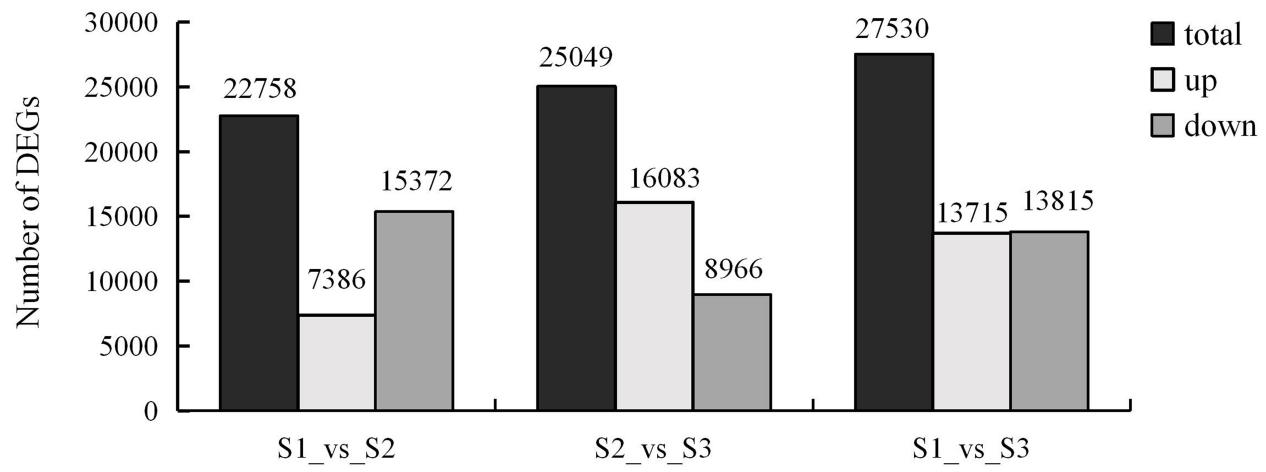


**Fig. S2** Comparison of the number of DEGs between two stages. Two different stages of development with total differentially expressed genes (black), up-regulated genes (light gray), and down-regulated genes (dark gray).


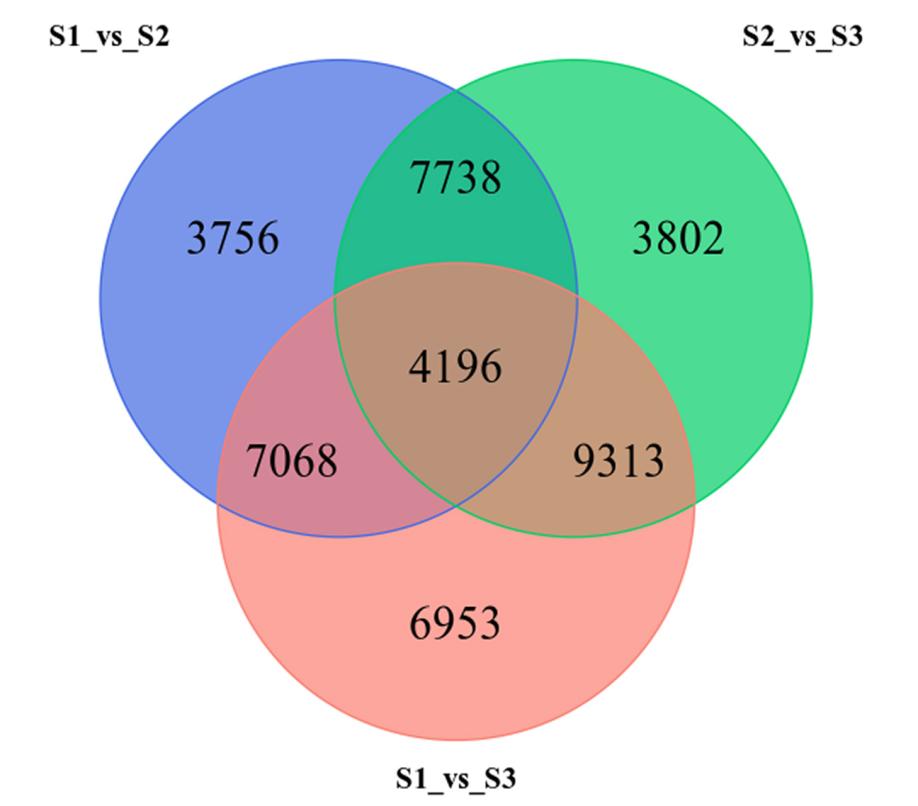


**Fig. S3** Venn map of DEGs among different stages.


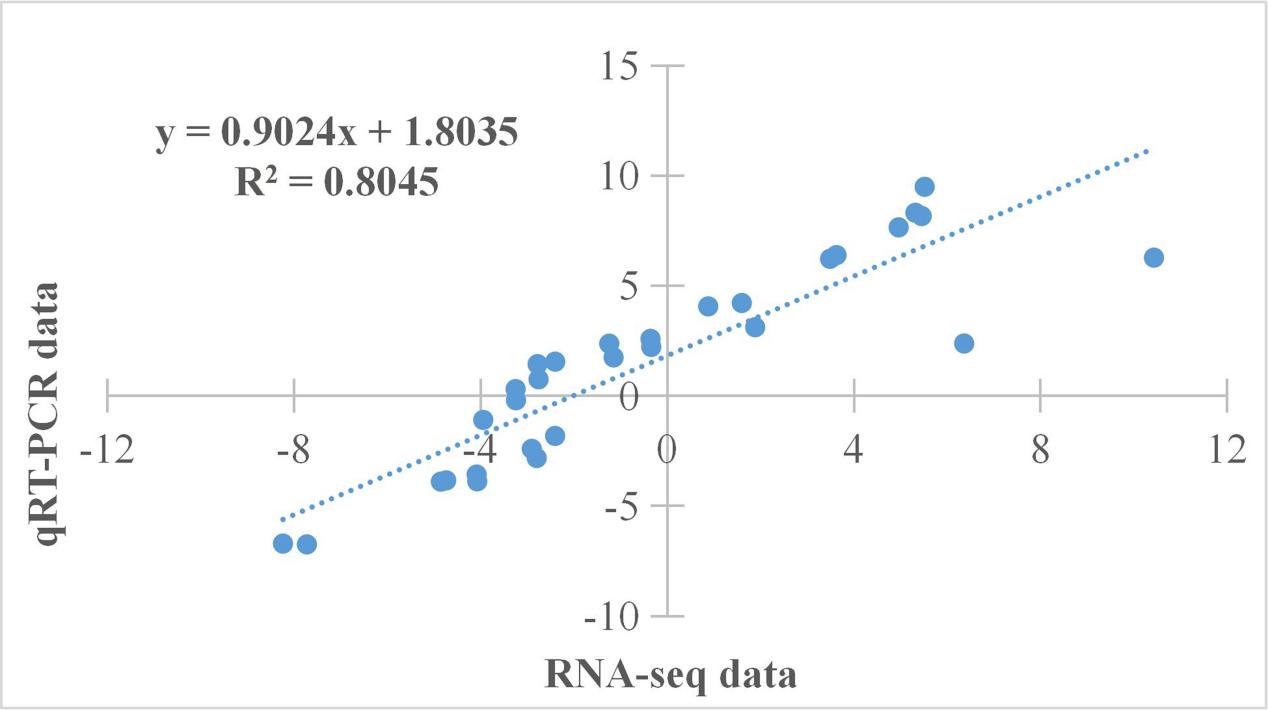


**Fig. S4** Validation analysis of transcriptome sequencing data.


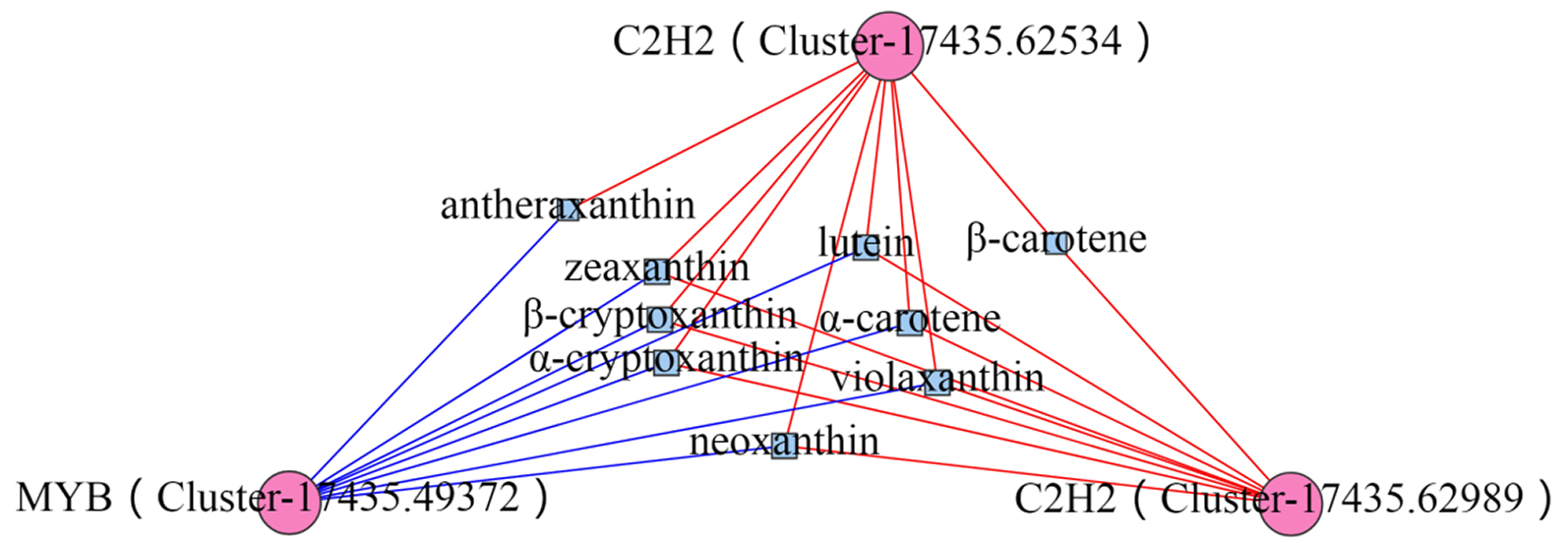


A.


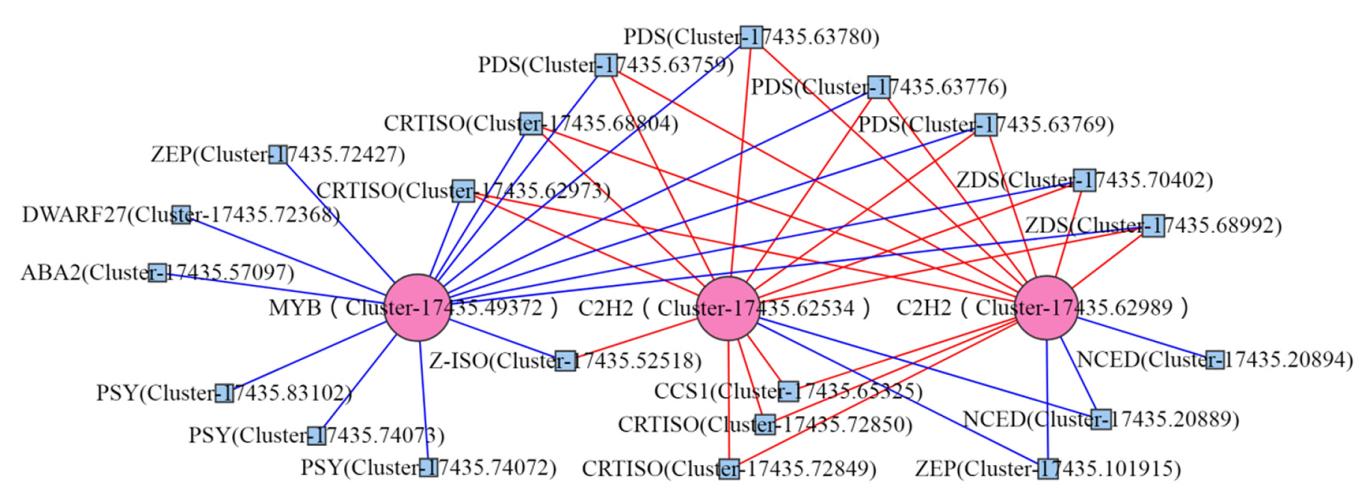


B.

**Fig. S5** Correlation network diagram of TFs with DAMs and DEGs. **A** Correlation between TFs and DAMs. **B** Correlation between TFs and DEGs. The circular shapes represent TFs, square shapes represent DAMs/DEGs; Red lines indicate positive correlations, while blue lines indicate negative correlations.
